# Comparative Analysis of the Nutritional Quality of *Zizania latifolia* Cultivars Harvested in Different Growing Seasons

**DOI:** 10.3390/foods13010030

**Published:** 2023-12-21

**Authors:** Guixian Hu, Xue Li, Aiping Lai, Yan Liu, Yu Zhang, Junhong Wang, Suling Sun, Jiahong Zhu, Mengfei Yang

**Affiliations:** 1 Institute of Agro-Product Safety and Nutrition, Zhejiang Academy of Agricultural Sciences, Hangzhou 310021, China; hugx_shiny@163.com (G.H.); laiaiping2022@163.com (A.L.); liuxiaoyan_ly@126.com (Y.L.); zhyu7711@163.com (Y.Z.); wangjunhong@zaas.ac.cn (J.W.); sunsuling123@126.com (S.S.); zjnky2011@126.com (J.Z.); 2 Key Laboratory of Information Traceability for Agricultural Products, Ministry of Agriculture and Rural Affairs of China, Hangzhou 310021, China; 3 Food Safety Key Laboratory of Zhejiang Province, Hangzhou 310021, China; 4 Provincial Key Laboratory of Characteristic Aquatic Vegetable Breeding and Cultivation, Jinhua Academy of Agricultural Sciences, Jinhua 321000, China; banmatus@foxmail.com

**Keywords:** water bamboo shoots, vegetable, nutrition, variety, harvest season

## Abstract

*Zizania latifolia* (*Z. latifolia*) is a popular aquatic vegetable with various nutrients in south China, but little is known about its cultivars and growing seasons in terms of the nutritional components. This work aims to characterize the nutrients of five *Z. latifolia* cultivars in different growing seasons. The results showed that *Z. latifolia* samples differed in terms of chemical parameters, which were significantly affected by variety, growing season, and their interaction. Zhejiao No. 8, harvested in the autumn, stood out with the highest levels of vitamin C. Tangxiajiao and Zhejiao No. 1 contained the highest values of total soluble solids, reducing sugar, soluble proteins, and amino acids. Significant differences were observed between the autumn *Z. latifolia* and spring samples; the former were of higher quality than the latter based on hierarchical clustering analysis and principal component analysis. Moreover, total amino acids (TAA) and glutamic acid (GLU) were selected as the key indicators for *Z. latifolia* comprehensive quality by multiple linear regression analysis. This study provides essential information on *Z. latifolia* quality characteristics corresponding to cultivars and growing seasons, which lays the foundation for promoting the quality improvement of *Z. latifolia* scientifically.

## 1. Introduction

*Zizania latifolia* (*Z. latifolia*, Turcz, fam. Poaceae), named Jiaobai or gausun, has been cultivated as a delicious and popular aquatic vegetable in East Asian countries, such as China, Russia, Japan, and Korea, for more than 2000 years [1]. The edible swollen culm of *Z. latifolia* is formed by the smut fungus *Ustilago esculenta* infection [2]. *Z. latifolia* is rich in healthy nutrients, including dietary fiber, protein, soluble sugar, vitamins, minerals, and so on [3,4]. Additionally, *Z. latifolia* has been used in traditional Chinese medicine due to its diverse biological effects, such as suppressing hyperlipidemia and oxidative stress, reducing blood glucose levels, improving insulin resistance, and ameliorating obesity [5,6]. In China, *Z. latifolia* has become the second most-cultivated aquatic vegetable, with more than 70,000 ha of cultivation areas and a value of 3 billion yuan each year [2,7,8]. As production increases, *Z. latifolia*, with both sensorial and nutraceutical characteristics, has become a new demand on the consumer market.

The accumulation of chemicals in different *Z. latifolia* is influenced by multiple factors. A previous study revealed that exogenous plant hormones could regulate the growth of *Z. latifolia* and have a significant effect on its chemical composition [4]. Some research also found that the dynamic changes of active ingredients in *Z. latifolia* under different storage conditions [3,9]. Although these studies have investigated its physicochemical indexes, few of them have paid attention to the production of nutrient compounds in *Z. latifolia* based on the cultivars and growing seasons. 

Variety is generally one of the main factors considered in evaluating vegetable quality, as chemical compositions may vary depending on genotype. Vast studies have verified that the cultivar can modify the accumulation of compounds in crop plants, such as pepper, olive, and blackcurrant fruit [10,11,12], and so on. For *Z. latifolia*, a number of cultivars have been bred over the past several decades, with single-season and double-season being the two main types [7]. The single-season crop plant can be harvested only once a year in the fall, and the double-season crop plant can be harvested twice in the fall and the following spring or summer, respectively. Different cultivars of *Z. latifolia* showed diverse phenotypic characteristics in terms of color, morphology, texture, and flavor quality [13]. The accumulation complexity of compounds in *Z. latifolia* samples from different cultivars remains a challenge for the development of characteristic *Z. latifolia*. 

In addition to cultivars, the harvest season is another principal factor in the quality formation of agriculture plants. As a typical seasonal product, the tea samples harvested in spring, summer, and autumn have been found to have remarkable variations in their metabolic profiles, like phenolic compounds, purine alkaloids, and amino acids [14,15], which demonstrated the profound impact of the harvest season on the chemical components of tea. Previous studies have also reported that growing seasons significantly influence the contents of carotenoids and phenolic compounds in red lettuce and biquinho pepper, respectively [10,14]. These studies suggest that endogenous chemical substances in crop plants are highly related to the growing season, which could result in a discrepancy in environmental conditions. Environment played a key role in the production of *Z. latifolia* since Ustilago esculenta, inducing the formation of the edible gall, needed a specific environmental condition to stay active [16]. In addition, the climatic conditions of the two growing seasons for double-season *Z. latifolia* are quite different, which may exacerbate the fluctuation in the contents of key compounds in *Z. latifolia*. Hence, it deserves systematic research through the comparison of homogeneous *Z. latifolia* under different harvest seasons. 

Food quality is a complex concept because the components in it may be related to each other and contribute a different weight to the product quality characteristics [17]. However, at present, most studies evaluate the quality of *Z. latifolia* only by a simple comparison of the attributes (e.g., texture, color, components). These approaches are straightforward and valuable, but they may ignore the compositional complexity of *Z. latifolia* and the possible interactions between the compounds. Multivariate statistical analysis is the discipline of extracting information from multi-dimensional data sets by using mathematical and statistical methods. The advantages of the method are that it can analyze large samples with a huge amount of data, mine the internal relationship between parameters, and intuitively display the variation of samples [15]. Multivariate statistical analysis techniques, such as Principal component analysis (PCA), hierarchical cluster analysis (HCA), and correlation analysis, have been widely used to classify and evaluate comprehensive food quality [10,17,18]. Therefore, these methods could be used as an effective solution to find the key traits related to *Z. latifolia* quality.

In view of this, the aims of this study are as follows: (1) quantify the main physicochemical indicators in *Z. latifolia* samples, including moisture, vitamin C, fiber, soluble protein, reducing sugar, total soluble solids, and amino acids; (2) disclose the nutrient content diversity of *Z. latifolia* samples from different varieties and growing seasons; and (3) explain the comprehensive quality of *Z. latifolia* by using multivariate statistical analysis. This study is of interest to provide basic information on the accumulation characteristics of the main components in the five major *Z. latifolia* cultivars harvested in different growing periods.

## 2. Materials and Methods

### 2.1. Plant Material

**Figure 1:**
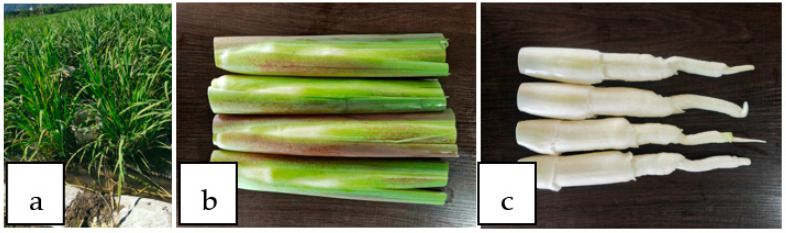
The plant of *Z. latifolia* (**a**), swollen gall with shell (**b**), and swollen gall without shell (**c**).

A total of 85 samples of *Z. latifolia* were collected from Huangyan District, Taizhou City, Zhejiang Province (121°20′ E, 28°69′ N), which was one of the main *Z. latifolia*-producing regions in China. These samples included one single-season plant (single-season plant Tangxiajiao: TJ) and four double-season cultivars (Zhejiao No. 1: ZJ1, Zhejiao No. 3: ZJ3, Zhejiao No. 7: ZJ7, and Zhejiao No. 8: ZJ8) (Table S1). The single-season samples were harvested in October 2021 and 2022 (season 1: from April to October in 2021, season 3: from April to October in 2022), respectively. The double-season samples were harvested in the fall of 2021, the spring and autumn of 2022 (season 1: from July to October in 2021, season 2: from October 2021 to April 2022, and season 3: from July to October in 2022), respectively. The double-season cultivars can be separated into spring *Z. latifolia* and autumn *Z. latifolia* according to the harvest season. A total of 2 kg of *Z. latifolia* with a shell were collected for each sample. All the samples had the same maturity (fresh stems expose about 1 cm of white parts from the shell) and no damage. The swollen galls were sent to the laboratory, and then they were cut and grinded with 6 short pulses of 1 min with a homogenizer (CR-001XS, Kormes, Zhongshan, China) immediately until they yielded a fine and homogeneous pulp. The samples were stored in a freezer at −20 °C. The plant *Z. latifolia*, swollen gall with and without shell, is shown in Figure 1. 

### 2.2. Determination of Moisture, Fiber, Total Soluble Solids, Reducing Sugar, Soluble Protein, and Vitamin C

The moisture was determined according to the Chinese National Official Standard (CNOS) GB 5009.3-2016 (Determination of moisture in food) [19]. Briefly, 2.0 g of sample was dried to a constant weight in an oven at 101~105 °C. Moisture was expressed as grams per 100 g of sample on fresh matter. 

Fiber was assayed using CNOS GB/T 5009.10-2003 (Determination of crude fiber in vegetable foods) [20]. A brief, accurately measured sample was placed in a conical bottle with 100 mL of 1.25% sulfuric acid. The mixture was boiled in water for 2 h. After cooling to room temperature, the extraction was filtrated by a sand core funnel, and the funnel and residue were dried to a constant weight in an oven at 105 °C. The result was expressed as grams per 100 g of sample on fresh matter. 

Total soluble solid (TSS) was measured by a WYT-4 handheld refractometer (Quanzhou Zhongyou Instrument Co., Ltd., Guangzhou, China) throughout the sample juice. The result was expressed as %.

Following the method described by CNOS GB 5009.7-2016 (National Food Safety Standard Determination of reducing sugar in foods) [21], reducing sugar (RS) content was determined by titration with an alkaline copper tartrate solution, using methylene blue as the indicator, and the result was expressed as g 100 g^−1^ fresh weight. 

Soluble protein (SP) and Vitamin C (VC) contents were assayed according to the previous method [22], and the results were expressed as g 100 g^−1^ and mg 100 g^−1^, respectively.

### 2.3. Determination of Amino Acid Contents 

Amino acids were analyzed according to the previous method [23]. Briefly, 1 g of sample was hydrolyzed by 6 M HCl (10 mL) in a closed-vessel digestion system at 110 °C for 24 h. The sample was analyzed by an amino acid analyzer equipped with cation-exchange chromatography and ninhydrin post-column derivatization (S433D SYKAM, Germany). Approximately 16 amino acids were determined, including aspartic acid (ASP), threonine (THR), serine (SER), glutamic acid (GLU), proline (PRO), glycine (GLY), alanine (ALA), valine (VAL), methionine (MET), isoleucine (ILE), leucine (LEU), tyrosine (TYR), phenylalanine (PHE), histidine (HIS), lysine (LYS), and arginine (ARG). Total amino acids (TAA) were calculated by adding 16 amino acids. The results were expressed as 100 g^−1^ of fresh weight. 

### 2.4. Data Analysis 

The SPSS statistics software package (Version 29; IBM, Armonk, New York, NY, USA) was used for statistical analysis. The data were subjected to variance analysis to test the effects of variety, growing season, and their interactions. And then the least significant difference (LSD) tests (*p* ˂ 0.05) were performed to determine the statistically significant differences among the five cultivars in different growing seasons. Hierarchical clustering heatmap analysis and correlation analysis were performed using the tools in Hiplot Pro (http://hiplot.com.cn/ (accessed on 4 November 2023)), a comprehensive web service for biomedical data analysis and visualization. 

Multiple linear regression (MLR) and Principal component analysis (PCA) were performed by the IBM SPSS Statistics 29 software package, and the PCA results were visualized by the Tutools platform (http://www.cloudtutu.com (accessed on 4 November 2023)), a free online data analysis website. In this study, the comprehensive quality of *Z. latifolia* was investigated by PCA, which was performed with the 23 measured nutritional variables. The score of the comprehensive evaluation was calculated with the following formulas:

F1 = α_11_ZX_1_ + α_21_ZX_2_ + … + α_231_ZX_23_

F2 = α_12_ZX_1_ + α_22_ZX_2_ + … + α_232_ZX_23_

F3 = α_13_ZX_1_ + α_23_ZX_2_ + … + α_233_ZX_23_

F4 = α_14_ZX_1_ + α_24_ZX_2_ + … + α_234_ZX_23_ (1)

where $\alpha =\frac{a}{\sqrt{\lambda }}$, a is the contribution rate (factor load) of each variable, λ is the eigenvalue, and Z is the standardized variable. According to Equation (1), the scores of the PCs were calculated and indicated. The ratio of the single PC contribution rate to the cumulative contribution rate was used as the weight to calculate the comprehensive evaluation scores of different *Z. latifolia*. According to Equation (2), the comprehensive evaluation model scores (F) were calculated.

(2) $$F=\frac{\lambda_{1}}{\lambda_{1}+\lambda_{2}+\lambda_{3}+\lambda_{4}}\times F_{1}+\frac{\lambda_{2}}{\lambda_{1}+\lambda_{2}+\lambda_{3}+\lambda_{4}}\times F_{2}+\frac{\lambda_{3}}{\lambda_{1}+\lambda_{2}+\lambda_{3}+\lambda_{4}}\times F_{3}+\frac{\lambda_{4}}{\lambda_{1}+\lambda_{2}+\lambda_{3}+\lambda_{4}}\times F_{4}$$

## 3. Results and Discussions

### 3.1. Difference Analysis for Physicochemical Parameters in Z. latifolia

#### 3.1.1. ANOVA for the Quality Parameters

In this study, 23 parameters were selected to assess the quality of *Z. latifolia* based on the reference [2]. Table S2 shows the results of the physicochemical indexes determined in 85 *Z. latifolia* samples. Moisture, VC, TSS, RS, SP, and fiber contents of various *Z. latifolia* were determined as 91.00–94.60%, 2.82–11.40 mg 100 g^−1^, 4.00–7.30%, 1.60–4.90 g 100 g^−1^, 0.78–1.69 g 100 g^−1^, and 0.7–1.3 g 100 g^−1^. In addition, 16 amino acids were detected in *Z. latifolia* samples. The individual amino acid mean values ranged from 0.001 to 0.22 g 100 g^−1^, and their total contents were from 0.60 to 1.38 g 100 g^−1^. Among them, ASP and GLU were two predominant amino acids accumulated in *Z. latifolia*, whereas MET was trace concentration. Similar physicochemical values of moisture, TSS, RS, and SP were also reported in other studies [2,13]. The variation coefficients of the sample’s VC, TSS, RS, SP, fiber, TAA, and 16 amino acids were between 0.12 and 0.40, indicating that these parameters fluctuated within a relatively wide range. The differential accumulation of chemical substances is responsible for the variation in taste and quality of *Z. latifolia*. 

**Figure 2:**
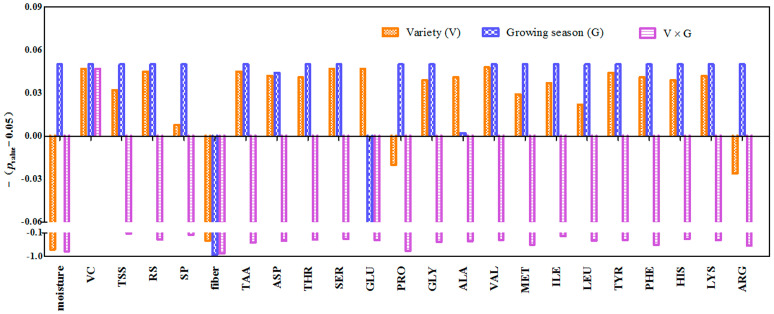
Effects of Variety, Growing season, and their interaction on each parameter in *Z. latifolia*. *p*_value_: significance of difference by ANOVA; −(*p*_value_ − 0.05) > 0: the effect had a significant difference; vice versa. Total soluble solid (TSS), reducing sugar (RS), soluble protein (SP), vitamin C (VC), aspartic acid (ASP), threonine (THR), serine (SER), glutamic (GLU), proline (PRO), glycine (GLY), alanine (ALA), valine (VAL), methionine (MET), isoleucine (ILE), leucine (LEU), tyrosine (TYR), phenylalanine (PHE), histidine (HIS), lysine (LYS), arginine (ARG), and total amino acids (TAA).

An analysis of variance (ANOVA) was applied to evaluate the effect of different factors such as variety (V) and growing season (G) on the contents of moisture, VC, TSS, RS, SP, fiber, and amino acids in *Z. latifolia*. The results are shown in Figure 2. Based on the *p* values, fiber content was not affected by the factors of cultivar, growing season, or their interaction (*p* > 0.05). A significant interaction (V × G) effect was only observed for VC. Meanwhile, the variety effect was significant for VC, TSS, RS, SP, TAA, ASP, SER, GLU, ALA, VAL, MET, ILE, LEU, TYR, PHE, HIS, and LYS. Moreover, all the variables were affected by the factor of growing season, with GLU being the only exception. The ANOVA results revealed that the values of the investigated parameters could be separately affected by cultivar and growing season, except for VC.

#### 3.1.2. VC Content Variations between Different Varieties and Growing Seasons

**Figure 3:**
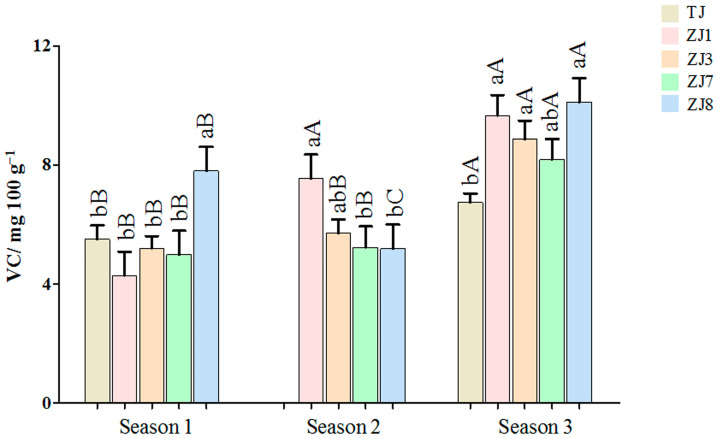
The Vitamin C (VC) contents of the five varieties of *Z. latifolia* in three growing seasons. Means followed by the same lowercase letter for cultivars and uppercase letter for growing season are not different according to the least significant difference tests at 5% of probability.

VC is one of the most important bioactive compounds in *Z. latifolia*, with antioxidant and antibacterial properties [24,25]. In addition, enhanced VC accumulation is beneficial to improve postharvest fruit quality and extend fruit shelf life [26]. In this study (Figure 3), VC content showed statistically significant differences (*p* < 0.05) between different cultivars and growing seasons. Among the cultivars in each growing season, ZJ8 obtained in seasons 1 (7.80 mg 100 g^−1^) and 3 (10.12 mg 100 g^−1^) showed the highest mean values of VC. The VC content of cultivar ZJ8 in autumn was more than 1.5 times that of the lowest variety in the same season. In the samples from season 2, the cultivar ZJ1 contained the highest average content of VC (7.55 mg 100 g^−1^) while ZJ8 presented the lowest mean value of VC (5.19 mg 100 g^−1^). Considering the growing season, significant differences (*p* < 0.05) were obtained among all the investigated cultivars in VC contents. The five cultivars in season 3 contained the highest mean values of VC compared to those of the same variety in season 1. ZJ8 in season 2 contained significantly lower VC amounts than those in season 1. However, lower levels of VC were found in season 1 compared with those in season 2 for the cultivar ZJ1 sample. Therefore, for VC accumulation in *Z. latifolia*, the effect of the growing season was more important than that of variety.

There is limited study on the variation of VC related to *Z. latifolia* cultivar and growing season. According to the available literature, crop cultivar clearly played a significant role in the content of VC [26]. Meanwhile, the growing season was another factor that affected the value of VC in pepper and blackcurrant fruit [11,26]. The variation of environmental conditions, such as temperature, rainfall, and solar radiation, may explain the influence of the growing season. The higher light intensity was positively related to the higher content of VC in vegetables [26]. Previous studies have also reported that the interaction between variety and growing season was observed for quality attributes such as phenolic compounds, carotenoids, and antioxidants in biquinho pepper and red lettuce [10,14]. In this study, *Z. latifolia* varieties reacted differently to the climate conditions of each growing season for VC accumulation. Among them, the mean concentration of ZJ8 in autumn was higher than that of the same season in the remaining cultivars. High concentrations of VC in autumn *Z. latifolia* may be one of the characteristics of ZJ8, which was a new cultivar certificated in 2020 in China.

#### 3.1.3. Effect of Variety and Growing Season on the Contents of Moisture, SP, TSS, and RS

The moisture content was the dominant component in fresh *Z. latifolia* (>90%). Growing season had a significant effect on its content (Figure 2), and the highest mean value of moisture (93.85%) was observed in the samples harvested in spring (season 2). This may be due to the lower temperature in spring. Meanwhile, the influences of *Z. latifolia* cultivar and growing season separately on the SP, TSS, and RS values were proved in this study (Figure 2). Protein is well known to be essential for human health. Soluble protein is a main component in *Z. latifolia*. The contents of SP in these *Z. latifolia* cultivars ranged from 1.15 g 100 g^−1^ to 1.33 g 100 g^−1^. As shown in Table 1, higher levels of SP were found in cultivars ZJ1 and TJ, while the lowest concentration of SP was presented in the ZJ8 cultivar. For the season factor (Table 1), levels of SP in spring samples (season 2) were lower than those in autumn (season 1). However, the contents of SP in autumn samples did not vary between the different cultivation years (2021–2022). Previous studies found a quadratic response between temperature and protein in soybeans; the protein content had a negative correlation with temperature between 14 and 20 °C and a positive correlation with temperature above 25 °C [27,28]. The differences in SP of *Z. latifolia* samples between the growing seasons could be due to climate change.

As shown in Table 1, the mean amounts of TSS between the different varieties ranged from 5.02% to 6.20%. The highest content of TSS was present in cultivar TJ, and ZJ3 had the lowest TSS concentration. When considering the season factor (Table 1), the spring samples (season 2) presented the lowest levels of TSS (4.52%) compared with the autumn samples (season 1, 5.02%). Significant differences in TSS were also observed between the different growing years (season 1 and season 3). The same results can be seen for the variable RS (Table 1). The mean contents of RS were within a range from 2.87 g 100 g^−1^ to 3.90 g 100 g^−1^ among the different varieties, for which TJ showed the highest RS value among the remaining cultivars. Samples harvested in spring had a significantly lower level of RS compared with those in autumn. A significant difference in the values of TSS and RS among different cultivars has been reported by a previous study [13]. Total soluble solids is a refractometric index that can be used to evaluate the sum of sugars, acids, and other minor nutrients in fruits [29,30]. Sugars are closely related to the sweet flavor of fruits and vegetables, their potential nutritive value, and consumer acceptance [23,31]. Therefore, the cultivar TJ may have a good taste as a vegetable. Except for the genetic/cultivar factor, previous studies reported a profound effect on fruit sugar accumulation by environmental factors, which suggested that higher temperature and season changes in irradiance could promote sugar accumulation in tomatoes [30,32]. The results of this study were consistent with the previous findings, where higher concentrations of TSS and RS were observed in autumn *Z. latifolia*, growing in conditions with a higher temperature and longer solar radiation compared with spring *Z. latifolia*. 

#### 3.1.4. Effect of Variety and Growing Season on the Values of Amino Acids

Amino acids are important for vegetable quality in terms of nutrition. As the basic unit of proteins, amino acids not only play a key role in plant physiology but are also beneficial to human health [33]. Some dietary amino acids act as antioxidants to scavenge free radicals; eight essential amino acids for humans must be obtained from food [34]. In this study, the tested amino acids included 7 essential amino acids (THR, VAL, MET, PHE, LYS, ILE, and LEU) and 9 conditionally essential amino acids (ASP, SER, GLU, PRO, GLY, ALA, TYR, HIS, and ARG). Multiple comparisons were performed for all the amino acids between the different cultivars and growing seasons, respectively (Table 2). When comparing among the cultivars, TJ and ZJ1 contained the highest concentrations of ASP, THR, SER, PRO, GLY, ALA, VAL, ILE, LEU, TYR, PHE, HIS, LYS, ARG, and total amino acids. The GLU value of ZJ1 (0.15 g 100 g^−1^) was the highest compared to those of other cultivars. As regards MET, the highest level was found in ZJ3 (0.01 g 100 g^−1^). Specifically, the cultivar ZJ8 showed the lowest contents of all the tested amino acids. Additionally, for the total content of essential amino acids, a difference was shown between the varieties TJ and ZJ8; the total conditionally essential amino acid contents were higher in TJ and ZJ1. The results meant that the amino acid contents of the *Z. latifolia* samples were affected by genetic traits. Regarding the season factor, it could be seen that there were lower levels of all the amino acids in the spring *Z. latifolia* samples (season 2) compared with the autumn samples (season 1). Significant differences were also observed for the contents of THR, GLY, ALA, VAL, MET, ILE, LEU, TYR, HIS, LYS, ARG, essential amino acids, and total amino acids between the different growing years (season 1 and season 3). The results indicated that the growing season had significant effects on the accumulation of amino acids in *Z. latifolia*, especially essential amino acids. Higher temperatures may be positively correlated with the accumulation of amino acids. Some researchers also found that genotype and environmental factors significantly influenced amino acid values in soybeans [27,28]. 

### 3.2. Multivariate Analysis

#### 3.2.1. Hierarchical Clustering Heatmap Analysis and Pearson’s Correlation Analysis

**Figure 4:**
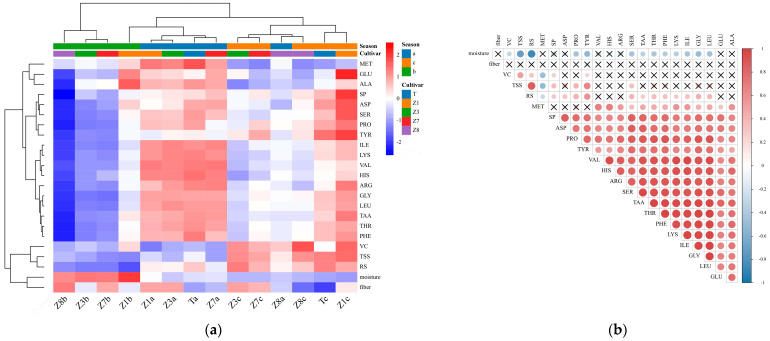
Hierarchical clustering heatmap analysis for the *Z. latifolia* samples (**a**) and Pearson’s correlations between the quality parameters (**b**). Total soluble solid (TSS), reducing sugar (RS), soluble protein (SP), vitamin C (VC), aspartic acid (ASP), threonine (THR), serine (SER), glutamic (GLU), proline (PRO), glycine (GLY), alanine (ALA), valine (VAL), methionine (MET), isoleucine (ILE), leucine (LEU), tyrosine (TYR), phenylalanine (PHE), histidine (HIS), lysine (LYS), arginine (ARG), and total amino acids (TAA). The a, b, and c corresponded to season 1, season 2 and season 3, respectively. Ta and Tc: tangxiajiao in season 1 and season 3, respectively; Z1a, Z1b and Z1c: Zhejiao No. 1 in season 1, season 2 and season 3, respectively; Z3a, Z3b and Z3c: Zhejiao No. 3 in season 1, season 2 and season 3, respectively; Z7a, Z7b and Z7c: Zhejiao No. 7 in season 1, season 2 and season 3, respectively; Z8a, Z8b and Z8c: Zhejiao No. 8 in season 1, season 2 and season 3, respectively. Red and blue colors indicated positive and negative correlations, respectively. No significant differences (*p* ≥ 0.05) were indicated with the multiplication sign.

Hierarchical clustering heatmap analysis is a helpful tool to provide a holistic overview of the distribution of the detected parameters and cluster the samples based on their similarity. We applied this analysis method to show the relative contents of 23 parameters in the samples from different varieties and seasons. A two-way HCA and its related heatmap diagram of the samples are shown in Figure 4a. The results showed that the tested samples, regardless of the cultivar, could be basically classified into spring *Z. latifolia* and autumn *Z. latifolia* based on the growing seasons. The result indicated that the growing seasons may contribute more to the quality of *Z. latifolia*. The samples harvested in spring (season 2) were grouped in cluster one, which was distinguished by higher contents of moisture and fiber and lower contents of reducing sugar, TSS, VC, SP, 16 amino acids, and total amino acids. The second cluster included samples harvested in autumn (season 1 and season 3, except ZJ 1 in season 2). The second group could be divided into two sub-groups by the different cultivation years. The first one was identified by the higher values of 16 amino acids, total amino acids, and SP; the other one was characterized by a higher content of VC, TSS, and RS and the lower concentrations of moisture and fiber. From the relationship between the analysis parameters, SP, 16 amino acids, and total amino acids were highly related to each other, as were VC, TSS, and RS. TSS and RS are the main parameters related to the flavor, and VC, SP, and amino acids are important nutrients. According to the characteristics of the second group, the members of this group can be the priorities for consumption of *Z. latifolia*.

The correlation was used to examine the inherent relationships between the chemical variables of *Z. latifolia*. These indicators could affect product quality together. The correlations of *Z. latifolia* physicochemical parameters are shown in Figure 4b. Firstly, moisture was significantly negatively correlated with VC, TSS, RS, and the amino acids, including PRO, TYR, SER, TAA, THR, PHE, LYS, ILE, GLY, and LEU. This suggested that the increased moisture content was not conducive to the accumulation of nutrients in *Z. latifolia*. Fiber content was not related to any parameter. Furthermore, VC, TSS, RS, and SP values showed statistically positive correlations with each other, indicating that flavor, taste, and nutrition were not independent factors but reciprocal effects. In addition, VC and TSS contents had weak correlations with the most amino acids. RS content was correlated with the amino acids, except for VAL, HIS, ARG, GLU, and ALA. Finally, SP and all the amino acids (except MET) showed significant positive correlations with each other. Unlike other amino acids, MET was negatively correlated with VC, TSS, and RS and not related to the SP value. Among the parameters, SP and TAA were significantly correlated with the most quality parameters, suggesting that they could be the ideal indicators for *Z. latifolia* quality evaluation. 

#### 3.2.2. Comprehensive Evaluation by PCA and MLR

The correlation analysis in Section 3.2.1 showed that the correlation degrees between different physicochemical indicators were diverse, and there was a strong correlation between some chemical indicators. If all these collinear indicators are included in the evaluation method, it will lead to information redundancy. Therefore, PCA was utilized to extract key physicochemical indicators, visualizing the differentiation between samples. PCA is a valuable tool to exploit more information on the variables mainly influencing the character of the sample by reducing the dimension of the original data [17]. In this study, two principal components (PCs) were selected based on the eigenvalue and total contribution rate of PCs [17], which had an eigenvalue greater than 1 and a total variance of 86.71% (Table 3). The results indicated that the two PCs could summarize most of the information from the original 23 variables. Thus, they were focused on further analysis.

**Figure 5:**
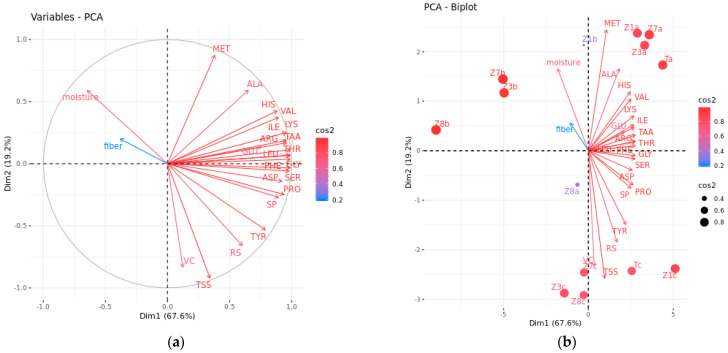
PCA loading plot (**a**) and biplot (**b**) in the main component of *Z. latifolia* samples at different cultivars and growing seasons. Red displayed a high cos2, and blue displayed a low cos2. Total soluble solid (TSS), reducing sugar (RS), soluble protein (SP), vitamin C (VC), aspartic acid (ASP), threonine (THR), serine (SER), glutamic (GLU), proline (PRO), glycine (GLY), alanine (ALA), valine (VAL), methionine (MET), isoleucine (ILE), leucine (LEU), tyrosine (TYR), phenylalanine (PHE), histidine (HIS), lysine (LYS), arginine (ARG), and total amino acids (TAA); tangxiajiao (TJ), Zhejiao No. 1 (ZJ1), Zhejiao No. 3 (ZJ3), Zhejiao No. 7 (ZJ7), and Zhejiao No. 8 (ZJ8).

In the component matrix (Table 3), the absolute value of the variable reflects the contribution of each parameter to the principal component [17]. The larger the absolute value, the closer the relationship, and vice versa. Based on the results, the contents of SP (X_5_), TAA (X_7_), ASP (X_8_),THR (X_9_), SER (X_10_), GLU (X_11_), PRO (X_12_), GLY (X_13_), VAL (X_15_), ILE (X_17_), LEU (X_18_), PHE (X_20_), HIS (X_21_), LYS (X_22_), and ARG (X_23_) mainly reflected PC1 (contribution rate of 67.552%), indicating that PC1 mainly reflected the nitrogen nutrient quality. PC2 accounted for 19.158% of the total variability, primarily correlating with VC (X_2_), TSS (X_3_), RS (X_4_), and MET (X_16_); therefore, PC2 mainly explained the antioxidant activity and sweet taste of *Z. latifolia*. In the loading plot, the cos^2^ value and distance to the correlation circle of the variable were used to estimate the representation quality of the variable [9]. Generally, the higher the cos^2^ value and the closer to the circle, the better the representation of the indicator on the factor map. In Figure 5a, cos^2^ values were represented from high to low by red to blue. A good interpretation of the variables was displayed in the two main components. THR and TAA were the most important parameters to interpret for PC1; TSS was the most perfectly represented variable for PC2. According to the biplot in Figure 5b, the content of TSS is highly positively correlated with RS and VC and negatively correlated with moisture. The result suggested that the sweet samples had higher VC content, and both the two parameters were higher in the samples from season 3. SP and all the tested amino acids had a close relationship with each other. In addition, the biplot results showed that the samples were grouped based on the harvest season. It may be caused by the temperature variation during the growing season. The autumn samples had better quality than the spring samples. These results were consistent with those obtained by Hierarchical clustering analysis and correlation analysis.

Generally, the quality feature of food is a complex and comprehensive concept that involves a variety of properties, such as sensory, physicochemical, nutritional, and functional aspects [35,36]. Therefore, the evaluation based on a single quality attribute is not intuitive and accurate. In vegetables and fruits, sugars, soluble proteins, amino acids, and VC are recognized as key quality compounds due to their sensory and nutritional attributes [37,38]. Nowadays, the internal quality with good taste and flavor is focused by consumers. According to the breeding characteristics and current market demand, a good *Z. latifolia* product for market consumption requires high RS, TSS, vitamin C, SP, and amino acids, as well as normal moisture content and low fiber. These indicators can be replaced by two PCs, and therefore, PCs can represent comprehensive *Z. latifolia* quality. 

In this part, the 85 samples evaluated could be divided into 14 groups based on their varieties and growing seasons. The final model evaluation scores of *Z. latifolia* were ranked in Table 4. The top five groups with high potential consumer preferences were Z1c, Ta, Z7a, Tc, Z3a, and Z1a, based on the final rank of comprehensive quality. It should be noted that the top five ranks were the samples obtained in autumn (seasons 1 and 3). The results indicated that the taste and nutrition of *Z. latifolia* harvested in the autumn were superior to those harvested in the spring. Generally, the temperature and total solar radiation time in the autumn in the Huangyan area were significantly higher than those in the spring. In terms of variety, TJ, ZJ1, and ZJ7 showed the highest comprehensive quality during the whole harvesting season.

Multiple linear regression (MLR) is a statistical technique that uses several explanatory variables to predict the outcome of a response variable. In this study, MLR with the stepwise regression method was applied to further screen the key traits and quantitatively determine their effects on the comprehensive quality of *Z. latifolia*. In this study, dependent variables were comprehensive scores, while the chemical parameters were controlled variables. The results of stepwise regression for the parameters are shown in Table S3. The regression equation was: Z = −98.029 + 117.308 × TAA − 116.700 × GLU; R^2^ was 0.998. TAA and GLU had a positive and a negative contribution to the quality of *Z. latifolia*, respectively. The result indicated that TAA and GLU significantly affected the quality of *Z. latifolia* (*p* < 0.01), suggesting that they could be the ideal indicators for *Z. latifolia* quality evaluation.

## 4. Conclusions

In summary, the results showed that a wide diversity exists within the *Z. latifolia* samples collected from different cultivars and growing seasons. The intricate impact of variety, growing season, and their interaction has been corroborated by important quality-related compounds of *Z. latifolia*, such as VC, RS, SP, TSS, and amino acids. HCA and PCA revealed that the comprehensive quality of autumn *Z. latifolia* was better than that of spring *Z. latifolia.* Based on the results of the MLR, the variables TAA and GLU have the most important effect on *Z. latifolia* comprehensive quality. This study will be helpful in the design of guidelines for agricultural practices and high-quality product selection.

## Figures and Tables

**Table 1:** Moisture, soluble protein (SP), total soluble solid (TSS), and reducing sugar (RS) at different cultivars and growing seasons. Significant differences (*p* ≤ 0.05) were indicated with the different lowcase letters. Tangxiajiao (TJ), Zhejiao No. 1 (ZJ1), Zhejiao No. 3 (ZJ3), Zhejiao No. 7 (ZJ7), and Zhejiao No. 8 (ZJ8).

Cultivars	Moisture (%)	SP (g 100 g^−1^)	TSS (%)	RS (g 100 g^−1^)
TJ	92.34 ± 0.61 a	1.33 ± 0.16 a	6.20 ± 0.78 a	3.90 ± 0.49 a
ZJ1	92.98 ± 0.96 a	1.35 ± 0.15 a	5.51 ± 1.13 b	2.97 ± 0.89 b
ZJ3	92.91 ± 0.76 a	1.25 ± 0.12 b	5.02 ± 0.81 c	2.95 ± 0.83 b
ZJ7	92.94 ± 0.70 a	1.25 ± 0.10 ab	5.10 ± 0.95 c	2.87 ± 0.74 b
ZJ8	92.82 ± 0.78 a	1.15 ± 0.18 b	5.62 ± 0.92 d	2.94 ± 0.71 b
Harvest Seasons	Moisture (%)	SP (g 100 g^−1^)	TSS (%)	RS (g 100 g^−1^)
Season 1	92.56 ± 0.34 b	1.29 ± 0.11 a	5.02 ± 0.51 b	3.25 ± 0.35 b
Season 2	93.85 ± 0.36 a	1.15 ± 0.16 b	4.52 ± 0.37 c	2.01 ± 0.31 c
Season 3	92.24 ± 0.55 c	1.34 ± 0.14 a	6.54 ± 0.48 a	3.94 ± 0.44 a

**Table 2:** Contents of 16 amino acids of *Z. latifolia* in five cultivars harvested in the spring and autumn seasons between 2021 and 2022. Tangxiajiao (TJ), Zhejiao No. 1 (ZJ1), Zhejiao No. 3 (ZJ3), Zhejiao No. 7 (ZJ7), and Zhejiao No. 8 (ZJ8); season 1 (October 2021), season 2 (April 2022), season 3 (October 2022); aspartic acid (ASP), threonine (THR), serine (SER), glutamic (GLU), proline (PRO), glycine (GLY), alanine (ALA), valine (VAL), methionine (MET), isoleucine (ILE), leucine (LEU), tyrosine (TYR), phenylalanine (PHE), histidine (HIS), lysine (LYS), arginine (ARG), essential amino acids (EAA), conditionally essential amino acids (CEAA), and total amino acids (TAA). Units: g 100 g^−1^. Significant differences (*p* ≤ 0.05) were indicated with the different lowercase letters.

Amino Acid Composition	Cultivars				
	TJ	ZJ1	ZJ3	ZJ7	ZJ8
ASP	0.158 ± 0.03 a	0.156 ± 0.017 a	0.134 ± 0.02 b	0.137 ± 0.02 b	0.123 ± 0.03 b
THR	0.053 ± 0.01 a	0.052 ± 0.005 ab	0.048 ± 0.01 c	0.048 ± 0.01 bc	0.044 ± 0.01 c
SER	0.061 ± 0.01 a	0.062 ± 0.008 a	0.054 ± 0.01 b	0.053 ± 0.01 b	0.050 ± 0.01 b
GLU	0.123 ± 0.02 b	0.148 ± 0.019 a	0.124 ± 0.02 b	0.120 ± 0.02 bc	0.108 ± 0.02 c
PRO	0.051 ± 0.01 a	0.050 ± 0.005 ab	0.045 ± 0.01 c	0.045 ± 0.01 bc	0.043 ± 0.01 c
GLY	0.057 ± 0.01 a	0.057 ± 0.007 a	0.052 ± 0.01 b	0.051 ± 0.01 b	0.047 ± 0.01 b
ALA	0.070 ± 0.01 a	0.074 ± 0.007 a	0.069 ± 0.01 a	0.068 ± 0.01 ab	0.062 ± 0.01 b
VAL	0.064 ± 0.01 a	0.064 ± 0.008 ab	0.060 ± 0.01 bc	0.058 ± 0.01 cd	0.052 ± 0.01 d
MET	0.009 ± 0.00 bc	0.010 ± 0.003 ab	0.011 ± 0.00 a	0.009 ± 0.00 bc	0.008 ± 0.00 c
ILE	0.047 ± 0.01 a	0.046 ± 0.006 ab	0.043 ± 0.01 bc	0.042 ± 0.01 cd	0.038 ± 0.01 d
LEU	0.085 ± 0.01 a	0.084 ± 0.009 ab	0.077 ± 0.01 bc	0.076 ± 0.01 bc	0.070 ± 0.01 c
TYR	0.038 ± 0.01 a	0.036 ± 0.006 a	0.031 ± 0.00 b	0.032 ± 0.01 b	0.030 ± 0.01 b
PHE	0.051 ± 0.01 a	0.050 ± 0.005 ab	0.046 ± 0.01 c	0.046 ± 0.01 bc	0.043 ± 0.01 c
HIS	0.033 ± 0.01 a	0.033 ± 0.003 a	0.031 ± 0.01 a	0.031 ± 0.00 ab	0.028 ± 0.00 b
LYS	0.077 ± 0.01 a	0.076 ± 0.009 ab	0.072 ± 0.01 bc	0.068 ± 0.01 cd	0.063 ± 0.01 d
ARG	0.056 ± 0.01 a	0.058 ± 0.007 a	0.052 ± 0.01 ab	0.052 ± 0.01 ab	0.045 ± 0.01 b
EAA	0.387 ± 0.06 a	0.360 ± 0.08 ab	0.357 ± 0.06 ab	0.346 ± 0.06 ab	0.318 ± 0.05 b
CEAA	0.648 ± 0.09 a	0.625 ± 0.14 a	0.593 ± 0.09 ab	0.588 ± 0.08 ab	0.536 ± 0.09 b
TAA	1.035 ± 0.14 a	1.056 ± 0.101 a	0.950 ± 0.14 b	0.934 ± 0.14 bc	0.854 ± 0.14 c
Amino Acid Composition	Harvest Seasons				
	Season 1	Season 2		Season 3	
ASP	0.147 ± 0.02 a	0.121 ± 0.02 b		0.154 ± 0.03 a	
THR	0.053 ± 0.00 a	0.042 ± 0.01 c		0.051 ± 0.01 b	
SER	0.060 ± 0.01 a	0.047 ± 0.01 b		0.059 ± 0.01 a	
GLU	0.130 ± 0.01 a	0.116 ± 0.02 b		0.125 ± 0.03 ab	
PRO	0.049 ± 0.01 a	0.040 ± 0.00 b		0.050 ± 0.01 a	
GLY	0.059 ± 0.01 a	0.043 ± 0.01 c		0.055 ± 0.01 b	
ALA	0.073 ± 0.01 a	0.068 ± 0.01 b		0.066 ± 0.01 b	
VAL	0.071 ± 0.01 a	0.049 ± 0.01 c		0.059 ± 0.01 b	
MET	0.014 ± 0.00 a	0.009 ± 0.00 b		0.006 ± 0.00 c	
ILE	0.050 ± 0.01 a	0.035 ± 0.00 c		0.044 ± 0.01 b	
LEU	0.087 ± 0.01 a	0.065 ± 0.01 c		0.081 ± 0.01 b	
TYR	0.033 ± 0.00 b	0.027 ± 0.00 c		0.039 ± 0.01 a	
PHE	0.051 ± 0.01 a	0.041 ± 0.01 b		0.049 ± 0.01 a	
HIS	0.036 ± 0.00 a	0.027 ± 0.00 c		0.030 ± 0.00 b	
LYS	0.081 ± 0.01 a	0.061 ± 0.01 c		0.072 ± 0.01 b	
ARG	0.059 ± 0.01 a	0.044 ± 0.01 c		0.053 ± 0.01 b	
EAA	0.407 ± 0.04 a	0.301 ± 0.04 c		0.362 ± 0.05 b	
CEAA	0.646 ± 0.06 a	0.533 ± 0.09 b		0.631 ± 0.09 a	
TAA	1.054 ± 0.10 a	0.834 ± 0.12 c		0.993 ± 0.14 b	

**Table 3:** The loading of each variable in the principal components. Total soluble solid (TSS), reducing sugar (RS), soluble protein (SP), vitamin C (VC), aspartic acid (ASP), threonine (THR), serine (SER), glutamic (GLU), proline (PRO), glycine (GLY), alanine (ALA), valine (VAL), methionine (MET), isoleucine (ILE), leucine (LEU), tyrosine (TYR), phenylalanine (PHE), histidine (HIS), lysine (LYS), arginine (ARG), and total amino acids (TAA).

Parameters	Component Matrix	Component Matrix
	PC1	PC1
Moisture (X1)	−0.646	0.590
VC (X2)	0.124	−0.833
TSS (X3)	0.342	−0.920
RS (X4)	0.603	−0.660
SP (X5)	0.896	−0.271
Fiber (X6)	−0.381	0.200
TAA (X7)	0.989	0.113
ASP (X8)	0.923	−0.141
THR (X9)	0.991	0.068
SER (X10)	0.981	−0.059
GLU (X11)	0.757	0.137
PRO (X12)	0.940	−0.248
GLY (X13)	0.986	−0.034
ALA (X14)	0.652	0.592
VAL (X15)	0.896	0.373
MET (X16)	0.381	0.872
ILE (X17)	0.958	0.188
LEU (X18)	0.985	0.067
TYR (X19)	0.789	−0.533
PHE (X20)	0.987	0.036
HIS (X21)	0.883	0.425
LYS (X22)	0.957	0.250
ARG (X23)	0.959	0.168
Eigenvalue	15.537	4.406
Contribution rates (%)	67.552	19.158

**Table 4:** Principal component scores after standardization and ranking comparison among *Z. latifolia* samples. Ta and Tc: tangxiajiao in season 1 and season 3, respectively; Z1a, Z1b and Z1c: Zhejiao No. 1 in season 1, season 2 and season 3, respectively; Z3a, Z3b and Z3c: Zhejiao No. 3 in season 1, season 2 and season 3, respectively; Z7a, Z7b and Z7c: Zhejiao No. 7 in season 1, season 2 and season 3, respectively; Z8a, Z8b and Z8c: Zhejiao No. 8 in season 1, season 2 and season 3, respectively.

Sample	F1	F2	F (Score)	Rank
Ta	16.65	3.50	13.75	2
Tc	9.70	−4.91	6.47	6
Z1a	10.96	4.80	9.6	5
Z1b	−1.04	4.32	0.14	7
Z1c	19.44	−4.82	14.08	1
Z3a	12.60	4.31	10.77	4
Z3b	−18.87	2.36	−14.17	12
Z3c	−5.38	−5.82	−5.47	11
Z7a	13.64	4.74	11.67	3
Z7b	−19.14	2.93	−14.26	13
Z7c	−0.96	−4.97	−1.85	8
Z8a	−2.45	−1.39	−2.22	10
Z8b	−34.12	0.85	−26.39	14
Z8c	−1.04	−5.90	−2.11	9

## Data Availability

Data are contained within the article.

## Supplementary Materials

The following supporting information can be downloaded at: https://www.mdpi.com/article/10.3390/foods13010030/s1. Table S1: Information on the cultivar, growing season, and number of samples; Table S2: Physicochemical parameters determined in *Z. latifolia*; Table S3: Results of stepwise regression for various quality parameters.
