# Supplementary material for: Comparative Analysis of the Nutritional Quality of Zizania latifolia Cultivars Harvested in Different Growing Seasons
Source: Foods. 2023 Dec 21;13(1):30. doi: 10.3390/foods13010030 (PMC10778467; doi:10.3390/foods13010030)
Supplement: Supplementary file 1 [file foods-13-00030-s001.zip › foods-2728569-supplementary.pdf]

Supplementary Materials

Table S1 Information of cultivar, growing season and number of samples. Growing season: single season cultivar (TJ) was from April to October, double season cultivars (ZJ1, ZJ3, ZJ7, ZJ8) were from July to April of the following year. Season 1 (TJ): from April to October 2021, Season 3 (TJ): from April to October 2022, season 1 (ZJ1, ZJ3, ZJ7, ZJ8): from July to October 2021, season 2 (ZJ1, ZJ3, ZJ7, ZJ8): from October 2021 to April 2022, season 3 (ZJ1, ZJ3, ZJ7, ZJ8): from July to October in 2022.

Cultivar	Harvest time	Growing season	Total samples
Tangxiajiao (TJ)	October 2021	Season 1	9
Zhejiao No.1 (ZJ1)			3
Zhejiao No.3 (ZJ3)			12
Zhejiao No.7 (ZJ7)			3
Zhejiao No.8 (ZJ8)			3
Zhejiao No.1 (ZJ1)	April 2022	Season 2	3
Zhejiao No.3 (ZJ3)			9
Zhejiao No.7 (ZJ7)			4
Zhejiao No.8 (ZJ8)			3
Tangxiajiao (TJ)	October 2022	Season 3	20
Zhejiao No.1 (ZJ1)			4
Zhejiao No.3 (ZJ3)			5
Zhejiao No.7 (ZJ7)			4
Zhejiao No.8 (ZJ8)			3

Table S2 Physicochemical parameters determined in *Z. latifolia*. Total soluble solid (TSS), reducing sugar (RS), soluble protein (SP), vitamin C (VC), aspartic acid (ASP), threonine (THR), serine (SER), glutamic (GLU), proline (PRO), glycine (GLY), alanine (ALA), valine (VAL), methionine (MET), isoleucine (ILE), leucine (LEU), tyrosine (TYR), phenylalanine (PHE), histidine (HIS), lysine (LYS), arginine (ARG), and total amino acids (TAA).

**Parameters**	**Minimum**	**Maximum**	**Mean**	**SD**	**CV**
Moisture (%)	91.00	94.60	92.72	0.77	0.01
VC (mg 100 g^{-1})	2.82	11.40	6.53	1.99	0.31
TSS (%)	4.00	7.30	5.55	0.99	0.18
RS (g 100 g^{-1})	1.60	4.90	3.27	0.83	0.26
SP (g 100 g^{-1})	0.78	1.69	1.28	0.15	0.12
Fiber (g 100 g^{-1})	0.70	1.30	0.88	0.12	0.14

TAA (g 100 g^{-1})	0.60	1.38	0.98	0.15	0.15
ASP (g 100 g^{-1})	0.08	0.22	0.14	0.03	0.18
THR (g 100 g^{-1})	0.03	0.07	0.05	0.01	0.14
SER (g 100 g^{-1})	0.03	0.08	0.06	0.01	0.15
GLU (g 100 g^{-1})	0.08	0.19	0.12	0.02	0.18
PRO (g 100 g^{-1})	0.03	0.07	0.05	0.01	0.15
GLY (g 100 g^{-1})	0.03	0.08	0.05	0.01	0.16
ALA (g 100 g^{-1})	0.04	0.09	0.07	0.01	0.13
VAL (g 100 g^{-1})	0.04	0.09	0.06	0.01	0.18
MET (g 100 g^{-1})	0.001	0.02	0.01	0.00	0.40
ILE (g 100 g^{-1})	0.03	0.06	0.04	0.01	0.17
LEU (g 100 g^{-1})	0.05	0.11	0.08	0.01	0.16
TRY (g 100 g^{-1})	0.02	0.05	0.03	0.01	0.19
PHE (g 100 g^{-1})	0.03	0.07	0.05	0.01	0.15
HIS (g 100 g^{-1})	0.02	0.05	0.03	0.01	0.16
LYS (g 100 g^{-1})	0.05	0.11	0.07	0.01	0.16
ARG (g 100 g^{-1})	0.03	0.08	0.05	0.01	0.19

Table S3 Results of stepwise regression for various quality parameters. glutamic acid (GLU), and total amino acids (TAA).

**Term**	**Coefficient**	**Standard Error**	**T-value**	**P-value**	**VIF**
Constant	-98.029	1.310	-74.838	0.000	\
TAA	117.308	2.351	49.900	0.000	3.079
GLU	-116.700	16.439	-7.099	0.000	3.079
Regression equation	Z = -98.029 + 117.308 × TAA - 116.700 × GLU				
R^2	0.998				
Durbin-Watson	2.743				
